# Prediction of Postoperative Complications for Patients of End Stage Renal Disease

**DOI:** 10.3390/s21020544

**Published:** 2021-01-14

**Authors:** Young-Seob Jeong, Juhyun Kim, Dahye Kim, Jiyoung Woo, Mun Gyu Kim, Hun Woo Choi, Ah Reum Kang, Sun Young Park

**Affiliations:** 1 Department of Future Convergence Technology, Soonchunhyang University, Asan-si 31538, Korea; bytecell@sch.ac.kr (Y.-S.J.); 20171484@sch.ac.kr (D.K.); jywoo@sch.ac.kr (J.W.); 2 Department of Big Data Engineering, Soonchunhyang University, Asan-si 31538, Korea; jjjuhyun@sch.ac.kr; 3 Department of Anesthesiology and Pain Medicine, Soonchunhyang University Hospital Seoul, Seoul 04401, Korea; rlaansrb1@schmc.ac.kr (M.G.K.); 127543@schmc.ac.kr (H.W.C.); 4 SCH Convergence Science Institute, Soonchunhyang University, Asan-si 31538, Korea

**Keywords:** postoperative complication, machine learning model, end stage renal disease, postoperative complications, feature selection

## Abstract

End stage renal disease (ESRD) is the last stage of chronic kidney disease that requires dialysis or a kidney transplant to survive. Many studies reported a higher risk of mortality in ESRD patients compared with patients without ESRD. In this paper, we develop a model to predict postoperative complications, major cardiac event, for patients who underwent any type of surgery. We compare several widely-used machine learning models through experiments with our collected data yellow of size 3220, and achieved F1 score of 0.797 with the random forest model. Based on experimental results, we found that features related to operation (e.g., anesthesia time, operation time, crystal, and colloid) have the biggest impact on model performance, and also found the best combination of features. We believe that this study will allow physicians to provide more appropriate therapy to the ESRD patients by providing information on potential postoperative complications.

## 1. Introduction

There have been many statistical studies aimed at discovering correlations or relationships between certain factors (i.e., features, variables) and post-operative adverse outcomes (e.g., mortality, respiratory failure). For example, Colin P. Dunn et al. conducted statistical tests to compare risk assessment tools for predicting adverse cardiac events in kidney transplant patients [1]. James P. Wick et al. proposed an assessment tool for predicting 6-month mortality after dialysis initiation [2]; they used a logistic regression (LR) model to find potential variables associated with 6-month mortality. In [3], the surgical complexity score was developed by checking the Area under the ROC Curve (AUC) with various settings of the LR model. Yue Li et al. found risk factors of post-operative cardiopulmonary complications by retrospective analysis of 653 lung-cancer surgery cases [4]. These studies commonly utilized the LR model, but the LR model was used as a measuring tool, not a predictor; in other words, their major contribution was to discover promising variables (or features) related to adverse outcomes, and the LR model was used as a verification tool. Some studies investigated first-hitting-time models for uncertainty analysis and clinical purposes (e.g., estimated time to infection for burn patients) [5,6]. As described in [7], although these statistical studies uncovered a large amount of useful information, they are still practically limited because they are not suitable for developing real-world applications (e.g., forecasting post-operative outcomes).

End stage renal disease (ESRD) is a loss of renal function for 3 or more months, and is the last stage of chronic kidney disease that requires dialysis or a kidney transplant to survive. Most of these patients have multiple comorbidities including anemia, cardiovascular disease, and diabetes mellitus. Considering their severe comorbidities, patients with ESRD have significantly high perioperative risks. Indeed, the literature consistently demonstrated a higher risk of mortality in ESRD patients compared with patients without ESRD, both in the cardiac and noncardiac perioperative periods [8,9,10,11,12,13,14]. Across various types of noncardiac surgery, patients with chronic kidney disease had two- to tenfold higher risks of postoperative death and cardiovascular events than those with normal kidney function [15,16,17,18]. Additionally, these patients have difficulties in terms of management for hemodynamic stability; this is because of their high morbidity and frailty. As such, the prediction of postoperative complications is important regarding perioperative management and reducing these complications. Methods to estimate individual risk are needed to provide individualized care and manage ESRD populations. Many mortality prediction models exist but they have shown deficiencies in model development (e.g., data comprehensiveness, validation) and in practicality. Therefore, we aim to design easy-to-apply prediction models for postoperative complications in ESRD patients. Postoperative adverse cardiac events are a major cause of morbidity and mortality in patients after non-cardiac surgery [19,20]. Therefore, predicting the risk is important in reducing these complications. The purpose of this study is to make a proper models for predicting postoperative major cardiac event (MACE) in ESRD patients undergoing general anesthesia.

Data-driven models are used in many fields such as face recognition, intent prediction of dialog systems, and speech recognition. The most widely-used data-driven models are logistic regression (LR), support vector machine (SVM) [21], decision tree, random forest (RF) [22], naive Bayes (NB), artificial neural network (ANN), and extreme gradient boosting (XGB) [23]). These machine learning models learn from data and have shown successful performance in various medical fields. For example, Katiuscha Merath et al. predicted patient risk of post-operative complications (e.g., cardiac, stroke, wound dehiscence, etc.) using a decision tree, and achieved about 0.74∼0.98 C-statistics [24]. In [25], ANN and LR models were used to predict post-operative complications (e.g., cardiac, mortality, etc.), and achieved about 0.54∼0.84 AUC with a 95% confidence interval (CI). Paul Thottakkara et al. compared four models (SVM, generalized additive model (GAM), NB, and LR) for risk prediction of some post-operative complications (e.g., acute kidney injury, severe sepsis) [26], and the SVM had the best results of 77.7∼85.0% accuracy with 95% CI. YiMing Chen et al. compared five models (SVM, RF, rotation forest (RoF), bayesian network (BN), and NB) to predict post-operative complications (e.g., wound infection, free flap infection, etc.), and found that the RF gave the best accuracy of 89.084% [27]. In [28], XGB was employed to predict complications after pediatric cardiac surgery and achieved about 0.82 AUC. Christine K. Lee et al. designed a deep neural network (DNN), which is an artificial neural network with many layers for prediction of post-operative mortality [29]; the proposed DNN consists of 4 layers with 300 nodes followed by ReLU activation functions [30]. In [31], to predict complications after bariatric surgery, the authors used three deep learning models: deep neural networks, convolutional neural networks (CNN) [32], and recurrent neural networks (RNN) [33]. Their dataset was extremely biased, so the three networks exhibited very poor sensitivities (e.g., 0.06∼0.23). Surprisingly, a recent study reported that using machine learning models were even more accurate than human clinicians [34]. In this paper, we adopt various promising machine learning models to predict postoperative MACE in ESRD patients undergoing general anesthesia.

Our contributions can be summarized as follows. First, as far as we know, this is the first study to predict post-operative MACE in patients with ESRD using various machine learning models. The machine learning models used in this paper are the most widely-used models for various tasks (e.g., image analysis, speech recognition, text analysis, and healthcare applications), so we compare the models experimentally and find the most effective one. Second, we suggest carefully designed feature groups, and examine how much impact each has on the performance. We implement a tool of natural language processing (NLP) to extract informative clues from preanesthetic assessment documents written by clinicians before surgery. We also examine combinations of feature groups and try feature selection algorithms. Note that the features are collected from several different sources: devices (or sensors), electronic medical record (EMR), and documents. Therefore, this study aims at applying machine learning techniques to data collected from different sources to develop a medical application. We believe that the results of this study eventually help physicians to make better medical decisions so that the survival rate of patients will be increased.

## 2. Materials and Methods

### 2.1. Materials

This paper tackles the problem of predicting post-operative complications for patients with end stage renal disease (ESRD). We focus on major adverse cardiac events (MACE), making it a binary classification problem over two classes (MACE and Not-MACE). The MACE class means that the patient suffered from MACE within one month (i.e., 30 days) after any type of surgery. Other than the ESRD, we have no other conditions or constraints for the patients, so it can be said that we predict the potential post-operative MACE of any ESRD patients with any type of surgery.

**Figure 1:**
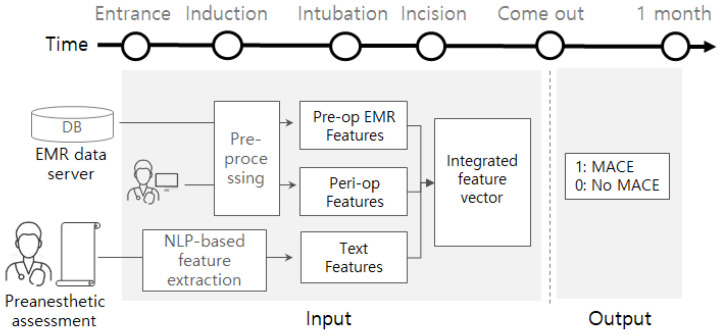
Input and output of the classifier.

This study was approved by the institutional review board of Soonchunhyang university Seoul hospital (approval No. SCHUH 2020-03-031). We collected data from electronic medical records (EMR) of the corresponding patients as well between the 1 March 2018 and 20 March 2020. The target patients are ESRD patients who underwent surgery under general anesthesia. There were not any other constraints (e.g., surgery type, age, disease history) of the patients. Data related to outcomes were obtained by submitting a batch data request to the Korean National Statistical Office (Microdata Integrated Service, on-demand) (https://mdis.kostat.go.kr) and electronic medical patient records. As depicted in Figure 1, pre-operational data is gathered from an EMR database server and pre-anesthetic assessment reports by the clinicians. Peri-operative data is obtained from the EMR database. Some attributes (e.g., EF, PFT, electrolyte) are collected from devices or sensors such as Ultrasound, spirometry, blood gas analyzer, or blood chemistry device. These data are monitored using attached devices or sensed using blood samples. We also collected preanesthetic assessment documents written by clinicians right before entrance into the surgery room.

### 2.2. Methods

The problem considered in this study is a binary classification. We adopt and compare several machine-learning models as a binary classifier. As shown in Figure 1, for a given patient, the machine-learning models commonly take a feature vector obtainable before or during the operation and are trained to predict its potential label (e.g., 1 or 0). Once we have a trained model, then the model analyzes the data for a new patient, helping physicians by providing information on how likely post-operative MACE is.

#### 2.2.1. Features

The input of the classification model is the integrated feature vector as represented in the center of Figure 1. It is obtained by concatenating three features that came from different sources: pre-op EMR features, peri-op features, and text features. The pre-op EMR features include demographic values (e.g., height, weight, sex, age, body mass index (BMI)), several pre-op evaluation results (e.g., EF, PFT), pre/post hemodialysis evaluations (e.g., Na, K, Cl), and comorbidities (e.g., hypertension, atrial fibrillation). By preprocessing, the categorical attribute ‘sex’ is converted into {0,1}${}^{1}$ where 0 and 1 represent male and female, respectively. Multi-valued attribute ‘PFT’ (e.g., 3.63(100)-2.48(104)-68) is separated into five smaller attributes (PFT1, PFT2, PFT3, PFT4, and PFT5 = [3.63, 100, 2.48, 104, 68]). Every comorbidity is converted into a binary attribute; if a patient suffered from hypertension before, then a binary attribute of hypertension is represented by 1, otherwise 0. If a particular attribute ‘A’ of the pre-op features has any missing values, then we define an additional attribute ‘A_missing‘ to denote the value of ‘A’ is missing or not. The peri-op features include anesthesia-related values (e.g., ASA, EM; emergency operation, anesthesia method), and other operation-related values (e.g., anesthesia time, operation time, infusion of crystalloid or colloid). Attribute ‘ASA’ is divided into two smaller attributes, ASA3 and ASA4; if ASA3 is 1, then the ASA of the patient is 3. As our target patients are ESRD, all patients were either of ASA3 or ASA4. The attribute ’anesthesia method’ is one of {volatile anesthesia, total intravenous anesthesia (TIVA)}, so it is converted into {0,1}${}^{1}$ where 0 and 1 imply volatile and TIVA, respectively. We also define an additional attribute if there are any missing values of a certain attribute of the peri-op features. The text features are generated by applying natural language processing (NLP) techniques to preanesthetic assessment documents, so it is obtainable before the operation.

The preanesthetic assessment is natural language text, so it is written without any template and there can be different texts for the same content; this is mainly the result of inconsistent usage of domain terms. For example, to denote ‘enlarged LA chamber size’, different people may use different terms such as ‘LA enlargement’ and ‘LAE’. Note that we need to extract features from the preanesthetic assessment document, and the features can be divided into two categories: (1) binary features and (2) numerical features. Both features are extracted through rule-based natural language processing; we design a set of rules to extract and normalize some keywords and numerical representations. About the binary features, we first define a set of target keywords as shown in Table 1, where the sample terms include synonyms or several different terms for the same keyword. For instance, the keyword ‘CAD’ may appear as different terms (e.g., CAD, coronary artery disease, coronary artery dz, and coronary artery stenosis), so these terms are listed in the sample terms. We design rules to detect these terms and normalize them to have the consistent term ‘CAD’. If the terms of ‘CAD’ appeared together with its level (e.g., CV1D, CV2D, CV3D), then it is regarded as a distinct keyword (e.g., CAD1, CAD2, CAD3). Given a list of $K_{binary}$ keywords, we will have binary features of {0,1}${}^{K_{binary}}$, where 1 implies the corresponding keyword appears in the preanesthetic assessment, and 0 means it does not.

For the numerical features, we also prepare a list of keywords and design rules to extract numerical values for the keywords. The list of numerical values are summarized in Table 2; for example, we extract two numerical values (e.g., TFT fT4 = 1.66, TFT T3 = 1.01) for the keyword ‘TFT’ which stands for thyroid gland function test. Note that this is the *information extraction* task in the NLP field. We implemented this information extraction process using a publicly available library ‘slotminer’ (https://github.com/bytecell/slotminer) that was originally designed for rule-based temporal information extraction [35]. We designed rules to detect and normalize the desired text patterns using the ‘slotminer’ library. If we obtain the numerical features $R^{K_{numerical}}$, this is concatenated with the binary features {0,1}${}^{K_{binary}}$; we eventually have a $K_{text}$ (i.e., $K_{numerical}+K_{binary}$) dimensional real-numbered vector.

Given the three features (e.g., pre-op EMR features $R^{K_{pre}}$, peri-op features $R^{K_{peri}}$, and text features $R^{K_{text}}$), we get the integrated feature vector $f\in R^{K}$ by concatenating those three features where $K=K_{pre}+K_{peri}+K_{text}$. Some elements (e.g., Age, Glucose, Egfr) of the integrated feature vector *f* are numerical and others (e.g., sex, COPD) are binary (i.e., 0 or 1). If a patient suffered from MACE within 30 days after the surgery, then its class label is 1, otherwise 0. In the perspective of the classification model, the input is the integrated feature vector *f* and the output is a scalar value t∈{0,1}.

#### 2.2.2. Models

We used several machine learning models: support vector machine (SVM), decision tree, random forest (RF), Gaussian naive Bayes (GNB), artificial neural network (ANN), logistic regression (LR), and extreme gradient boosting (XGB). SVM is known to be robust to outliers because its decision boundary is determined using only support vectors. The decision tree is simple and explainable, but is vulnerable to high variance. The random forest is kind of a bagging-based ensemble method, where it is known to overcome the limitations of high variance of decision trees without losing low bias. The Gaussian naive Bayes uses a Gaussian distribution to compute the probabilities of continuous values. The artificial neural network is a multi-layered perceptron that captures a high level of patterns beneath the input values. Extreme gradient boosting is an ensemble method utilizing a boosting algorithm, and is known to lower the variance and bias.

#### 2.2.3. Feature Groups

Instead of simply taking all attributes of *f* as an input, we design several feature groups, each of which is a subset of *f*, to examine which kind of features are more important. The feature groups are determined based on where or when the attributes came from. For example, as shown in Table 3, the *demographic* feature group includes basic patient information obtainable from the EMR database before entrance into the surgery room. The *history* feature group includes records about comorbidities from the EMR database before entering the surgery room. The *Electrolyte (pre&post hemodialysis)* feature group contains attributes of evaluation right before or after hemodialysis and is obtainable before surgery room entrance. The *text* feature group includes attributes extracted from the assessment documents as listed in Table 1 and Table 2. This feature group also has an additional binary feature ‘hasText’ that indicates whether there exists comments in the assessment or not; if the assessment document is empty, then it is 0, otherwise 1. The *PreEval* feature group includes results of pre-operational evaluation such as ejection fraction (EF), pulmonary function test (PFT), rapid plasma reagin (RPR), blood urea nitrogen (BUN), alanine aminotransferase (ALT), aspartate aminotransferase (AST), activated partial thromboplastin time (aPTT), international normalized ratio (INR), prothrombin time (PT), platelet (Plt), albumin (Alb), hematocrit (Hct), and hemoglobin (Hb). The *Anesthesia* feature group is related to the anesthesia process; some of its attributes (e.g., ASA values) are obtainable before surgery, but the others (e.g., method, EM) are determined during surgery. The *Operation* feature group is obtained during the operation. The feature dimensions of the above feature groups are 5, 32, 12, 77, 42, 4, and 4, respectively; so the total feature dimension K=176.

Other than the feature groups, we also adopt two feature selection algorithms: Recursive Feature Elimination (RFE) and K-best. If we want to select K features, then the RFE algorithm recursively eliminates non-promising features until K features remain. We use the RF model as an estimator for the RFE algorithm. The K-best algorithm selects K features according to scores computed using a particular function σ(f,c), where *f* indicates an arbitrary feature and *c* is a label. We use chi-squared stats for the function σ. In the experimental results of the next section, we exhibit the performance comparison between different feature groups and selected features.

## 3. Results

The total amount of data in the original dataset $D_{origin}$ is 3220. We found that the $D_{origin}$ is highly imbalanced; the ratio of MACE versus Not-MACE was almost 1:10. To settle this, we generated a balanced dataset by downsampling from $D_{origin}$, so we got a label ratio of 1:1 as a result. As the downsampling from $D_{origin}$ is performed randomly, we prepared three independently downsampled datasets, $D_{balanced^{1}}$, $D_{balanced^{2}}$, and $D_{balanced^{3}}$. Each of the three datasets is further divided into three sets: training, validation, and test sets, while approximately maintaining the balanced label ratio. The statistics of each dataset are summarized in Table 4.

We compared several machine learning models according to precision, recall, and F1 score. The models are implemented using scikit-learn package (https://scikit-learn.org/). Every model is trained using the training dataset, and its parameter is tuned using the validation dataset. The parameter settings of Table 5 are found by grid searching, which generally gave the best F1 score. The numerical attributes are scaled between 0 and 1, where the maximum and minimum values are extracted from the training dataset. We performed three independent experiments using the three downsampled datasets. Some machine learning models (e.g., ANN, RF) may give different results even if the same data are given, because of the parameter initialization method or ensemble process (e.g., random sampling).

Thus, we conducted 10 experiments for each of the sampled datasets ($D_{balanced^{1}}$, $D_{balanced^{2}}$, and $D_{balanced^{3}}$), and all results are thus a weighted average of 3 × 10 experiments.

**Figure 2:**
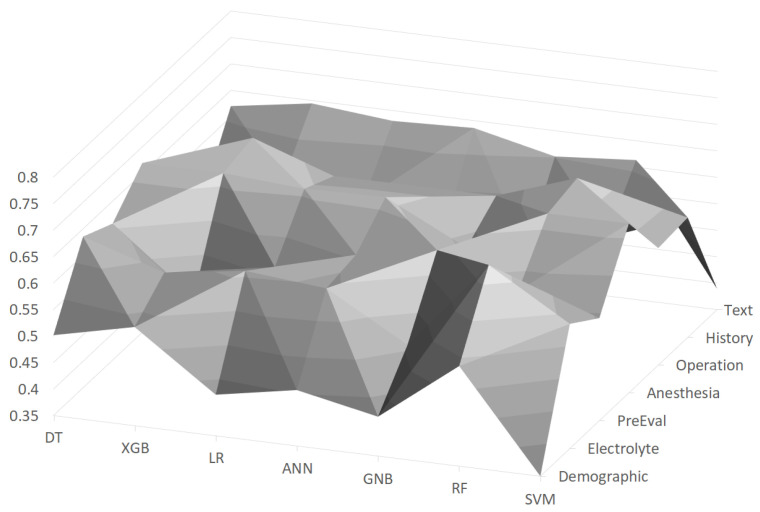
F1 scores using machine learning models with feature groups, where the vertical axis indicates scores.

In Figure 2, F1 scores using the machine learning models with feature groups are depicted as a surface chart, where the scores are distributed between about 0.4∼0.65. It turned out that the *Operation* feature group is the most important among the feature groups, and the random forest (RF) achieved the best F1 score of 0.684 with this feature group. On the other hand, the *Demographic* feature group generally turned out to be the worst feature group, as about half of the machine learning models gave low scores (e.g., lower than 0.45). The random forest (RF) was generally the best with almost all feature groups, and its F1 score ranged between 0.539 and 0.684.

We tried several combinations of feature groups as shown in Table 6. We can interpret the results from two perspectives: the perspective of feature combinations and the model-wise perspective. In terms of the feature combination perspective, we found that a combination of feature groups is much better than a single feature group. For example, the *Demographic* feature group alone was the worst among the feature groups, but using it with the *Electrolyte* feature group improved the F1 score about 0.7. We also found that a combination of more feature groups generally had better performance than that with fewer feature groups; for example, Demographic&Electrolyte&PreEval is better than Demographic&Electrolyte. However, using all feature groups did not help to improve the performance, and the most effective combination was Demographic&Electrolyte&PreEval&Anesthesia&Operation. In the model-wise perspective, the RF model generally gave the best performance with all combinations. The best F1 score of 0.797 was achieved by the RF model with the feature group combination Demographic&Electrolyte&PreEval&Anesthesia&Operation.

**Figure 3:**
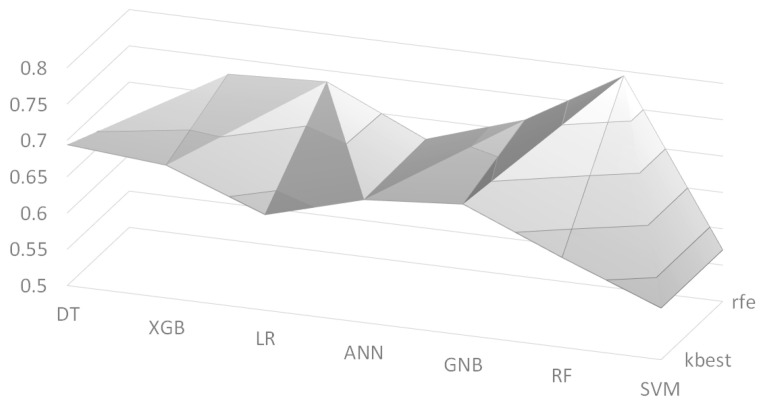
F1 scores using feature selection algorithms, where the vertical axis indicates scores.

We also employed two feature selection algorithms: Recursive Feature Elimination (RFE) and K-best. We set the number of selected features to K = 30, and its F1 scores are depicted in Figure 3 as a surface chart, where ‘kbest’ and ‘rfe’ indicate the K-best algorithm and the RFE algorithm, respectively. The RF model with the RFE algorithm had the best F1 score of 0.672 among the models. Table 7 shows the list of selected features by the two feature selection algorithms. As we performed three independent experiments, the list contains only features that appeared in at least two experiments. The two algorithms selected quite different features, but they commonly picked many features from two feature groups: *Operation* and *Anesthesia*. It is interesting that from the *Text* feature group, the ‘T3’ values of the thyroid gland function test (TFT) were commonly chosen by the two algorithms. This is consistent with the recent work [37] that found that low ‘T3’ values are associated with poor prognosis; although the target patients of this work are different, we can say that the feature selection algorithms picked reasonable features from the *Text* feature group. Another interesting point is that ‘hasText’ feature was picked by the K-best algorithm, where this feature simply indicates whether the preanesthetic assessment document has content or not. This can be explained that the clinicians usually write the preanesthetic assessment when there are any important issues regarding the patients so that ‘hasText’ could be an indicator of important issues.

## 4. Discussion

We found that the best feature group is *Operation* by experimental results. This is consistent with existing studies on predictors of postoperative cardiac events in patients undergoing non-cardiac surgery. For example, Myung Hwan Bae et al. investigated whether surgical parameters have prognostic value with respect to the development of a postoperative cardiac event [38]. They concluded that the surgical parameters, including surgery time and blood transfusion during surgery, were found to be independent predictors of postoperative MACE in patients undergoing non-cardiac surgery. Although their experiments included only 4.9% of renal insufficiency (defined as serum creatinine ≥2 mg/dL), this is consistent with our study that the *Operation* feature group is the most important for prediction of postoperative MACE in ESRD patients.

**Figure 4:**
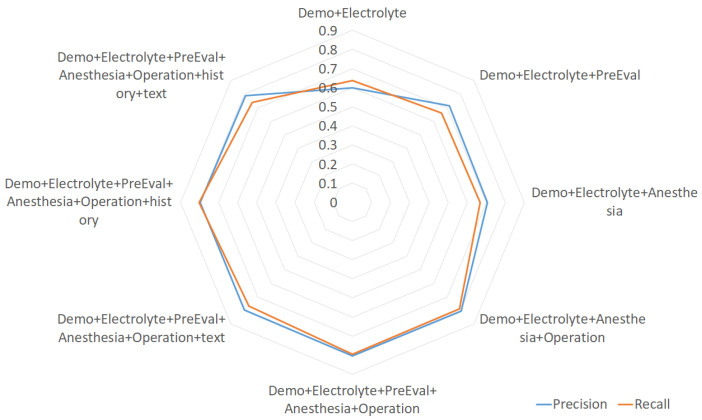
The radar chart of precision and recall of MACE class using random forest for feature group combinations.

Regarding the prediction model, one might argue that it is not very useful if it is not sensitive enough to MACE cases. Figure 4 shows the precision and recall values of the MACE class (i.e., label ‘1’) using the random forest (RF) model. We found that the RF model has a precision of 0.803 and a recall of 0.794, with the best feature combination of Demographic&Electrolyte&PreEval&Anesthesia&Operation. With this result, we might say that our prediction model is enough to inform physicians of the potential risk so that the patients will be provided with more appropriate therapy. As there is still plenty of room for improvement, we will keep investigating various methods to obtain better performance.

There are two ways of applying the results of this study to operations. First, the best model of this study (i.e., random forest) is installed onto a computer within the surgery room, where the model provides information about potential MACE right after the end of surgery. Physicians are provided with this information so that the patient will have better suited therapy. Of course, the information is just a predicted result (i.e., probability of potential MACE), so the appropriate medical decisions must be made by the physicians. Second, clinicians carefully check the features that we found important. For example, according to the experimental results, we found that *Operation* features are the most important and other particular features (e.g., anesthesia method, TFT T3) are informative to the prediction of postoperative MACE.

Although we achieved an F1 score of 0.797 and found some important features for the prediction of postoperative MACE, this study is limited because of the small data size. We are continually collecting more data, and plan to gather data from other sources (i.e., other hospitals); we believe that data collected from different sources will help to verify the generalization of our future model. We also expect that more data will improve the performance (i.e., greater F1 score) of data-driven prediction models.

## 5. Conclusions

In this paper, we applied various machine learning models to predict postoperative MACE in ESRD patients undergoing general anesthesia. We found that the random forest (RF) model gives the best F1 score of 0.797 with a particular combination of feature groups. We believe that our work will be helpful for physicians to make better medical decisions based on the information provided by our model. We will continue collecting data and investigate how to design a more effective prediction model. As some features extracted from preanesthetic assessment documents turned out to be promising, we will investigate how to use language models to extract more informative features from documents.

## Figures and Tables

**Table 1:** Target keywords of binary features.

Keyword	Sample Terms
COPD	asthma, bronchiectasis, emphysema
bedridden state	bedridden
cardiac arrest c ROSC	cardiac arrest, arrest, CPR
alcoholic hepatitis	alcoholic liver disease
LC	alcoholic liver cirrhosis, alcoholic LC, liver cirrhosis
IPF	pul.Fibrosis, pulmonary fibrosis
Anemia	IDA
MI	Myocardial infarction, AMI, NSTEMI, STEMI
PCI	coronary stent insertion, coronary stent, DES, BMS
Atherosclerosis	-
DVT	deep vein thrombosis
Carotid artery stenosis	Carotid a. stenosis
Cerebral atherosclerosis	-
ESRD on KT	KT state, KT transplant state, Kidney transplant state, ESRD s/p KT
hyperparathyroidism	-
Fatty liver	-
CAD	coronary artery disease, coronary artery dz, coronary artery stenosis
	levels: CV1D, CV2D, CV3D
Angina	Angina pectoris, Angina s/p PCI, unstable angina, variant angina
Depression	MDD, Depressive disorder, Depressive episode
VPC	PVC
APC	PAC
Right heart failure	right HF
CHF	HF, heart failure, congestive heart failure
MR	MVR, Mitral valve regurgitation, mitral regurgitation,
	MV regurgitation
Pulmonary hypertension	PH, Pul. HTN, Pulmonary HTN
Pulmonary embolism	PTE, pulmonary thromboembolism
TR	TVR, Tricuspid valve regurgitation, tricuspid regurgitation,
	TV regurgitation
HTN	hypertension
DM	diabetes mellitus
Cancer	ca.
CAG	-
	levels: C1VD, C2VD, C3VD, significant, normal
pneumonia	-
atelectasis	-
effusion	-
cardiomegaly	-
pulmonary edema	-
consolidation	-
ground glass opacity	-
EKG	-
PSVT	-
VPC	-
A. fib	AF, atrial fibrillation
tachycardia	-
bradycardia	-
Hypertensive heart disease	-
DCMP	-
ICMP	-
HHT	-
AR	AVR, Aortic valve regurgitation, Aortic regurgitation,
	AV regurgitation
PAOD	-
MVP	MV prolapse
Diastolic dysfunction	Pseudonormalization of LV filling pattern, E/A reverse,
	E/A < 1, Increased DT, Relaxation Abnormality
Concentric LVH	Concentric LV wall thickening, Concentric LV remodeling,
	Concentric LV hypertrophy
Eccentric LVH	Eccentric LV hypertrophy
HCMP	Hypertropic cardiomyopathy, Hypertropic CMP
LAE	Enlarged LA chamber size, LA enlargement, BAE
RAE	right atrial enlargement, RA enlargement,
	Enlarged RA chamber size
LVE	Enlarged LV chamber size, LV enlargement
RVE	RV enlargement, Enlarged RV chamber size,
	Enlarged RA & RV chamber size
Ischemic Heart Disease	IHD, CAD
Regional wall motion abnormality	RWMA
LV systolic dysfunction	LV dysfunction, hypokinesia, akinesia
RV dysfunction	-
A-fib	AF, atrial fibrillation
AS	aortic valve stenosis, rheumatic AS
AR	aortic regurgitation
MS	mitral valve stenosis, rheumatic MS, degenerative MS
	levels: mild, moderate, severe
uremic CMP	-
IVC plethora	-
pericardial effusion	-
pleural effusion	-

**Table 2:** Numerical values extracted from texts, where the bold parts are samples to be extracted.

Keyword	Sample
Glucose	Glucose: **50**
TFT	TSH/fT4/T3 0.30(L)/**1.66**(N)/**1.01**(N) (04/03)
aBGA	**7.48**-**39**-**101**-**29**-**98** (04/04)
EGFR	**44** (11/15)

**Table 3:** Feature groups.

Group Name	Features (Attributes)
Demographic	sex, age, weight, height, BMI
History	hypertension, artial fibrillation, coronary artery disease,
	angina pectoris, myocardial infarction, congestive heart failure,
	valvular heart disease, DCMP, asthma, COPD, GERD, hepatitis,
	interstitial lung disease, liver cirrhosis, viral carrier,
	fatty liver, HBV, HCV, alcoholic, autoimmune, acute kidney injury,
	chronic kidney injury, diabetes mellitus (DM), HbA1c, thyroid disease,
	myasthenia gravis, morbid obesity, epilepsy, cerebrovascular disease,
	cerebral aneurysm, dementia
Electrolyte (pre&post HD)	Na (pre), K (pre), Cl (pre), Na (post), K (post), Cl (post)
Text	All binary & numerical values listed in Table 1 and Table 2,
	hasText
PreEval	EF, PFT1, PFT2, PFT3, PFT4, PFT5, RPR, BUN, ALT, AST, aPTT,
	INR, PT, Plt, Ca, P, Alb, Hct, Hb, AntiHBs, HBsAg
Anesthesia	method, ASA3, ASA4, EM
Operation	anesthesia time, operation time, crystal, colloid

**Table 4:** Statistics of a sampled dataset.

	# of Data
Total	586
Train	475
Validation	53
Test	58

**Table 5:** Parameter settings of machine learning models.

Model	Setting
Decision tree	- Criterion: Gini
Random forest	- Number of estimators: 200
	- Criterion: Gini
Extreme gradient boosting	- Number of estimators: 200
	- Criterion: Mean squared error
Support vector machine	- Kernel: RBF
	- C: 1.0
Gaussian naive bayes	- Smoothing with $10^{-9}$
Artificial neural network	- Hidden layers: (100, 50, 25)
	- Activation functino: ReLu [30]
	- Solver: Adam [36]
	- Maximum # of iterations: 1000

**Table 6:** Performance with combinations of feature groups, where a tuple p/r/f indicates precision, recall, and f1 score.

Combination	DT	XGB	LR	ANN	GNB	RF	SVM	*F*
Demographic	0.560/	0.593/	0.541/	0.518/	0.631/	0.606/	0.531/	15.351 ***/
& Electrolyte	0.558/	0.593/	0.540/	0.516/	0.580/	0.605/	0.529/	13.058 ***/
	0.556	0.592	0.534	0.509	0.507	0.604	0.520	12.363 ***
Demographic	0.562/	0.654/	0.607/	0.611/	0.616/	0.699/	0.604/	15.730 ***/
& Electrolyte	0.561/	0.652/	0.603/	0.607/	0.580/	0.696/	0.603/	21.125 ***/
& PreEval	0.561	0.651	0.598	0.603	0.514	0.695	0.603	24.459 ***
Demographic	0.545/	0.646/	0.667/	0.600/	0.727/	0.695/	0.713/	63.257 ***/
& Electrolyte	0.545/	0.644/	0.661/	0.595/	0.655/	0.694/	0.690/	37.969 ***/
& Anesthesia	0.544	0.643	0.659	0.592	0.614	0.693	0.682	26.434 ***
Demographic & Electrolyte	0.704/	0.714/	0.711/	0.693/	0.770/	0.798/	0.671/	25.009 ***/
& Anesthesia	0.684/	0.710/	0.707/	0.689/	0.724/	0.797/	0.609/	41.059 ***/
& Operation	0.679	0.709	0.706	0.687	0.710	0.796	0.571	55.551 ***
Demographic & Electrolyte	0.672/	0.747/	0.667/	0.703/	0.770/	0.798/	0.671/	47.648 ***/
& PreEval & Anesthesia	0.668/	0.745/	0.667/	0.699/	0.724/	0.797/	0.609/	56.419 ***/
& Operation	0.665	0.745	0.666	0.697	0.710	0.797	0.571	67.391 ***
Demographic & Electrolyte	0.646/	0.683/	0.679/	0.645/	0.699/	0.797/	0.671/	58.158 ***/
& PreEval & Anesthesia	0.645/	0.682/	0.678/	0.642/	0.672/	0.795/	0.609/	65.421 ***/
& Operation & History	0.644	0.682	0.678	0.639	0.657	0.795	0.571	71.666 ***
Demographic & Electrolyte	0.624/	0.723/	0.679/	0.678/	0.738/	0.784/	0.671/	14.264 ***/
& PreEval & Anesthesia	0.622/	0.721/	0.678/	0.668/	0.713/	0.782/	0.609/	20.250 ***/
& Operation & Text	0.618	0.721	0.678	0.663	0.692	0.781	0.571	21.808 ***
Demographic & Electrolyte	0.624/	0.715/	0.656/	0.635/	0.707/	0.771/	0.671/	26.038 ***/
& PreEval & Anesthesia	0.623/	0.715/	0.655/	0.634/	0.695/	0.768/	0.609/	32.834 ***/
& Operation & History	0.621	0.715	0.654	0.633	0.688	0.768	0.571	39.371 ***
& Text								

*F*: *F* ratio, computed from the ANOVA table. (*** *p* < 0.001). Degrees of Freedom (*df*): 6 (between classifiers), 203 (within classifiers). Critical value *F*_*crit* of the test: 2.14345288.

**Table 7:** Selected features from different feature groups.

RFE	
Demographic	age, weight, height, BMI
Electrolyte	Na(pre), K(pre), Cl(pre), Na(post), K(post), Cl(post)
PreEval	EF, PFT1, PFT2, PFT3, PFT4, PFT5, BUN, Ca, P, Alb
Anesthesia	Method, ASA3
Operation	anesthesia time, operation time, crystal, colloid
History	HbA1c
Text	TFT values (fT4, T3)
**K-best**	
Demographic	-
Electrolyte	is missing?: Na(pre), K(pre), Cl(pre)
PreEval	-
Anesthesia	Method, ASA3, ASA4, EM
Operation	anesthesia time, operation time, crystal, colloid
History	angina pectoris, congestive heart failure, valvular heart disease, GERD,
	chronic kidney injury, thyroid disease
Text	EKG, DM, HTN, Cancer, TFT values (T3), LV systolic dysfunction,
	COPD, Angina, hasText

## Data Availability

Data available on request due to restrictions. The data presented in this study are available on request from the corresponding author. The data are not publicly available due to privacy and ethical issue.
